# The Transcription Factor DAF-16 is Essential for Increased Longevity in *C. elegans* Exposed to *Bifidobacterium longum* BB68

**DOI:** 10.1038/s41598-017-07974-3

**Published:** 2017-08-07

**Authors:** Liang Zhao, Yang Zhao, Ruihai Liu, Xiaonan Zheng, Min Zhang, Huiyuan Guo, Hao Zhang, Fazheng Ren

**Affiliations:** 1 Beijing Advanced Innovation Center for Food Nutrion and Human Health, College of Food Science and Nutritional Engineering, China Agricultural University, Beijing, 100083 China; 2 Key Laboratory of Functional Dairy, College of Food Science and Nutritional Engineering, China Agricultural University, Beijing, 100083 China; 3 Beijing Laboratory for Food Quality and Safety, China Agricultural University, Beijing, 100083 China; 4 Department of Food Science, Cornell University, Ithaca, NY 14853-7201 USA; 5 School of Food and Chemical Engineering, Beijing Technology and Business University, Beijing, 100048 China; 6 Beijing Higher Institution Engineering Research Center of Animal Product, College of Food Science and Nutritional Engineering, China Agricultural University, Beijing, 100083 China

## Abstract

The longevity-promoting benefits of lactobacilli were hypothesized as early as 1907. Although the anti-aging effects of lactic acid bacteria (LAB) have been observed in nematodes, rodents and humans for over a century, the mechanisms underlying the effects of probiotics on aging have rarely been assessed. Using the *Caenorhabditis elegans (C. elegans)* model, various studies have elucidated the role of different signaling cascades, especially the DAF-16 cascade, on lifespan extension by LAB. In this study, the mechanisms through which *Bifidobacterium longum* strain BB68 affects the longevity of *C. elegans* were assessed. The lifespan of nematodes increased by 28% after worms were fed BB68, and this extension of lifespan was completely lost in backgrounds containing a mutated DAF-16 gene. High levels of DAF-16 (in the daf-16 (mu86); muIs61 strain) nuclear accumulation and high expression of the SOD-3 gene (a DAF-16-specific target gene) were observed as a result of BB68 treatment. Immunofluorescence microscopy revealed that TIR-1 and JNK-1 are involved in the phosphorylation and activation of DAF-16. Thus, BB68 increased the longevity of nematodes by activating the TIR-1 – JNK-1 – DAF-16 signaling pathway, and the cell wall component of BB68 contributed to longevity.

## Introduction

Lactobacilli were hypothesized to be vital bacteria for promoting human health and longevity as early as 1907^1^. For a century, lactic acid bacteria (LAB), including bifidobacteria, have been commonly used as probiotic microorganisms due to their beneficial effects on the host. The anti-aging effects of these probiotics have been assessed in nematodes, rodents, and humans to evaluate improvements in immunosenescence in elderly animals^2–5^. Using the *Caenorhabditis elegans* (*C. elegans*) model, several studies have described the mechanisms through which LAB affects aging (Table 1), including mechanisms involving different longevity signaling pathways related to stress resistance and calorie restriction. The DAF-2/DAF-16, p38 MAPK/SKN-1, and JNK-1/DAF-16 signaling pathways were found to contribute to stress resistance, which contributes to prolonged lifespan, and AAK-2/DAF-16 might be associated with calorie restriction-dependent longevity (Table 1). Two major transcription factors, SKN-1 and DAF-16, may act as key longevity regulators in worms that respond to various LAB strains. The species/strain-specific mechanisms through which probiotics affect the longevity of nematodes were identified in previous studies. In particular, the role of DAF-16 and its signal transduction were found to be inconsistent in different studies.

**Table 1:** Summary of the effect of probiotics on the longevity of *C. elegans*.

Strain		Mechanism	References
*Lactobacillus gasseri* SBT2055	Increased median survival by 37%; higher pharyngeal pumping; enhanced resistance against intrinsic oxidative stress	Activated NSY-1–SEK-1–PMK-1; up-regulated the expression of SKN-1 and its target genes	16
*Weissella koreensis* KACC 11853 *Weissella cibaria* KACC 11845	Increased longevity; reduced pharyngeal pumping	Activated JNK-1–DAF-16 related to the stress response; Activated AAK-2–DAF-16 related to dietary restriction	17
*Lactobacillus salivarius* FDB89	Extended the mean life span by 11.9%; reduced reproductive capacity, pharyngeal pumping rate and growth; increased SOD and XTT reduction activity	Dietary restriction dependent (eat-2 and food gradient feeding assay)	18
*Bifidobacterium infantis* ATCC15697	Increased longevity in a dose-dependent manner; enhanced resistance to oxidative and heat stress	Activated PMK-1–SKN-1 to regulate phase 2 detoxification; DAF-2 is involved in SKN-1 regulation; the bacterial cell wall is a major effective constituent	15
*Lactobacillus rhamnosus* CNCM I-3690	Increased median survival by 3 days (from 15 days to 18 days); enhanced resistance to oxidative stress	Dependent (at least partially) on the DAF-2/DAF-16 signaling pathway; SKN-1 also involved	19

To study the anti-aging effects of probiotics, our laboratories isolated several LAB strains from centenarians living in an area with high longevity^6^ that may be responsible for the longevity of these individuals. Here, we show that *Bifidobacterium longum* BB68, a novel probiotic strain isolated from a centenarian living Bama, Guangxi^6^, increased lifespan in *C. elegans* by regulating the conserved innate immune signaling mediated by DAF-16. Thus, the cell wall components of BB68 might contribute to the longevity of nematodes.

In *C. elegans*, mitogen-activated protein kinase (MAPK) signaling and insulin-like signaling (ILS) regulate the process of aging and innate immunity^7, 8^. These pathways are well conserved across mammals and nematodes, which designates *C. elegans* as an ideal model for investigating the process of aging and immune regulation. In mammals, probiotic strains enhance host immunity by regulating the p38/MAPK signaling pathway^9^, suggesting that probiotics might affect the immunity of nematodes and aging through a homologous pathway. We previously reported that *Bifidobacterium longum* BB68, which was isolated from a centenarian, exerts potent activity in regulating immunity^6^; however, little is known about its effects on aging.

**Figure 1:**
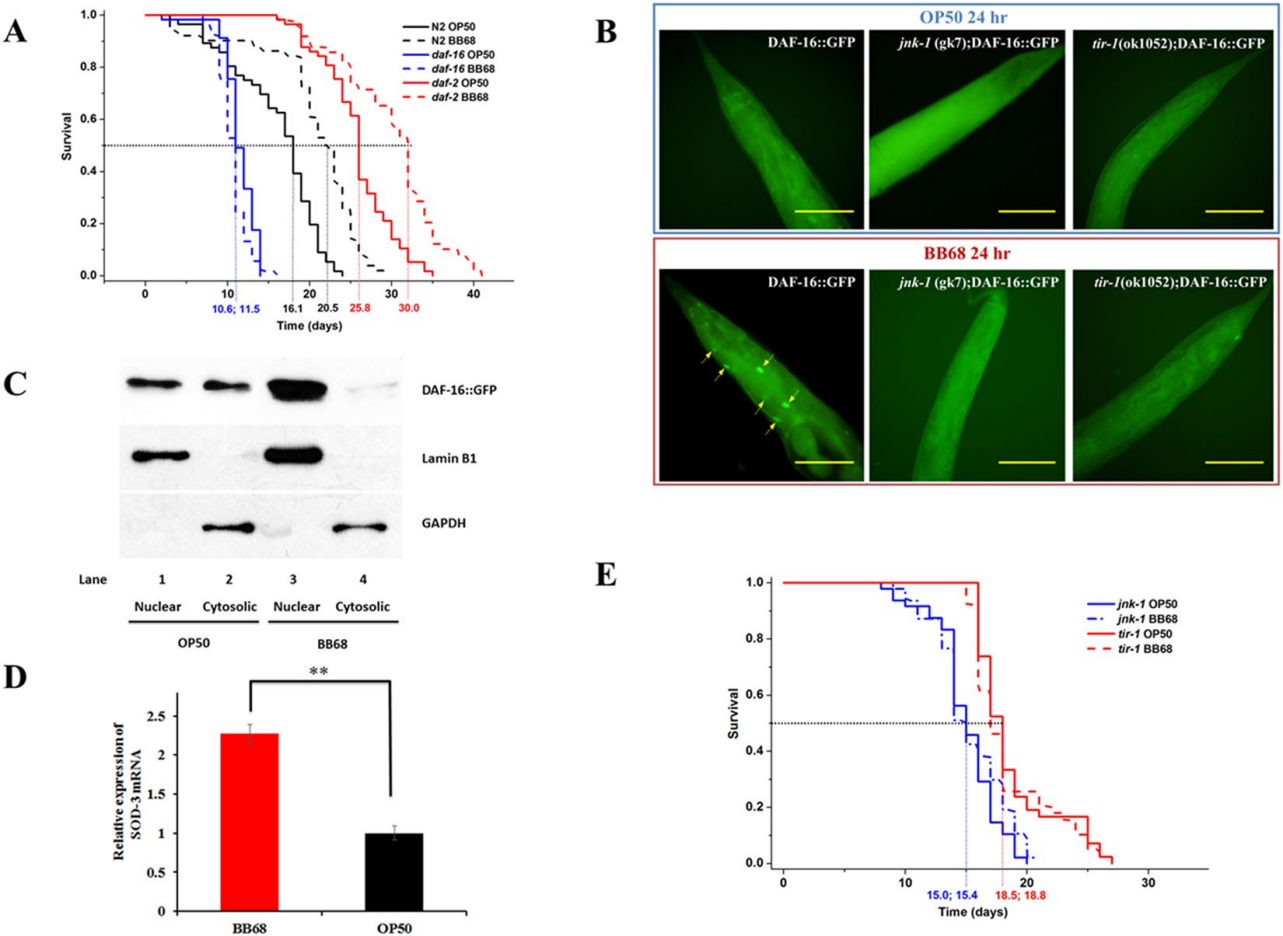
*Bifidobacterium longum* BB68 extends the lifespan of *C. elegans* by regulating TIR-1 – JNK-1 – DAF-16 signaling. (**A**) Feeding worms BB68 increased the lifespan of the N2 and *daf-2* strains, but not the *daf-16* strain. (**B**) Feeding DAF-16::GFP worms BB68 for 24 h activated DAF-16 (the arrows indicate increased nuclear accumulation of DAF-16), and lower levels of activated DAF-16 were detected in the presence of mutated *jnk-1* or *tir-1*. (**C**) Western blotting showed significant nuclear accumulation of DAF-16 protein in nematodes fed BB68 for 24 h. Lamin B1 was used as a nuclear reference, and GAPDH was used as a cytosolic reference. (**D**) An increase in SOD-3 gene expression level was observed in nematodes fed BB68 for 24 h. ^**^Indicates a significant difference (Student’s T-test, *p* < 0.01). The test was conducted in triplicate. (**E**) The presence of mutant *jnk-1* and *tir-1* completely eliminated the lifespan extension induced by BB68. Scale bar = 50 μm.

First, we tested whether BB68 could prolong the lifespan of *C. elegans*. Survival assays revealed that feeding BB68 to *C. elegans* could extend the lifespan of wild-type N2 organisms by 28% relative to the lifespan of those fed standard food, *Escherichia coli (E. coli)* OP50 (Fig. 1A; Table S1). Moreover, the lifespan extension by BB68 did not affect the nematodes’ pharynx pumping, body size, or reproductive ability (Table S2). These results are also supported by a previous study, in which *Bifidobacterium infantis* did not alter these indices^2^. The bacterial gradient concentration feeding assay (0.1–200 mg/plate) and *eat-2* (ad1116) mutant (eating defective mutant with the phenotype of calorie restriction) survival assays indicated that the BB68-mediated lifespan extension was independent of calorie restriction (Table S1; Fig. S1).

To investigate the role of ILS in the BB68-mediated longevity of *C. elegans*, DAF-16 and DAF-2, the key regulators of ILS, were evaluated. DAF-16, an FOXO transcription factor in *C. elegans*, controls the transcription of several antioxidant and chaperone genes that delay aging^10^. The lifespan assays showed that mutating DAF-16 (*daf-16* (mu86)) caused the BB68-mediated effect of prolonged lifespan to be completely lost (Fig. 1A; Table S1). This phenomenon indicated that such an extension is DAF-16 dependent. Using a DAF-16-GFP fusion reporter strain (*daf-16* (mu86); muIs61), we demonstrated that BB68 significantly increased the nuclear accumulation of DAF-16 by 56% (Fig. 1B; Table S3) relative to that induced by OP50. Western blotting also showed increased nuclear localization of DAF-16 protein (Fig. 1C). These results suggested the up-regulation of DAF-16 in response to BB68 treatment. To confirm the role of DAF-16 in BB68-induced longevity, the level of gene expression of SOD-3, one of the DAF-16-specific target genes, was determined by qPCR^11^. A significant increase in the expression of SOD-3 (2.27-fold compared to SOD-3 expression in worms fed OP50) was observed in worms fed BB68 for 24 h (Fig. 1D). Thus, we concluded that DAF-16 is essential for BB68-mediated lifespan prolongation.

DAF-2 is a human insulin receptor homolog upstream of DAF-16 in ILS, and mutations in DAF-2 decrease extensions in lifespan and resistance to pathogens by activating the nuclear translocation of DAF-16^8, 10^. Our results show that feeding BB68 to worms further prolongs the lifespan of *daf-2* (e1368) mutants (Fig. 1A; Table S1), indicating that the BB68-mediated regulation of DAF-16 is independent of DAF-2.

**Figure 2:**
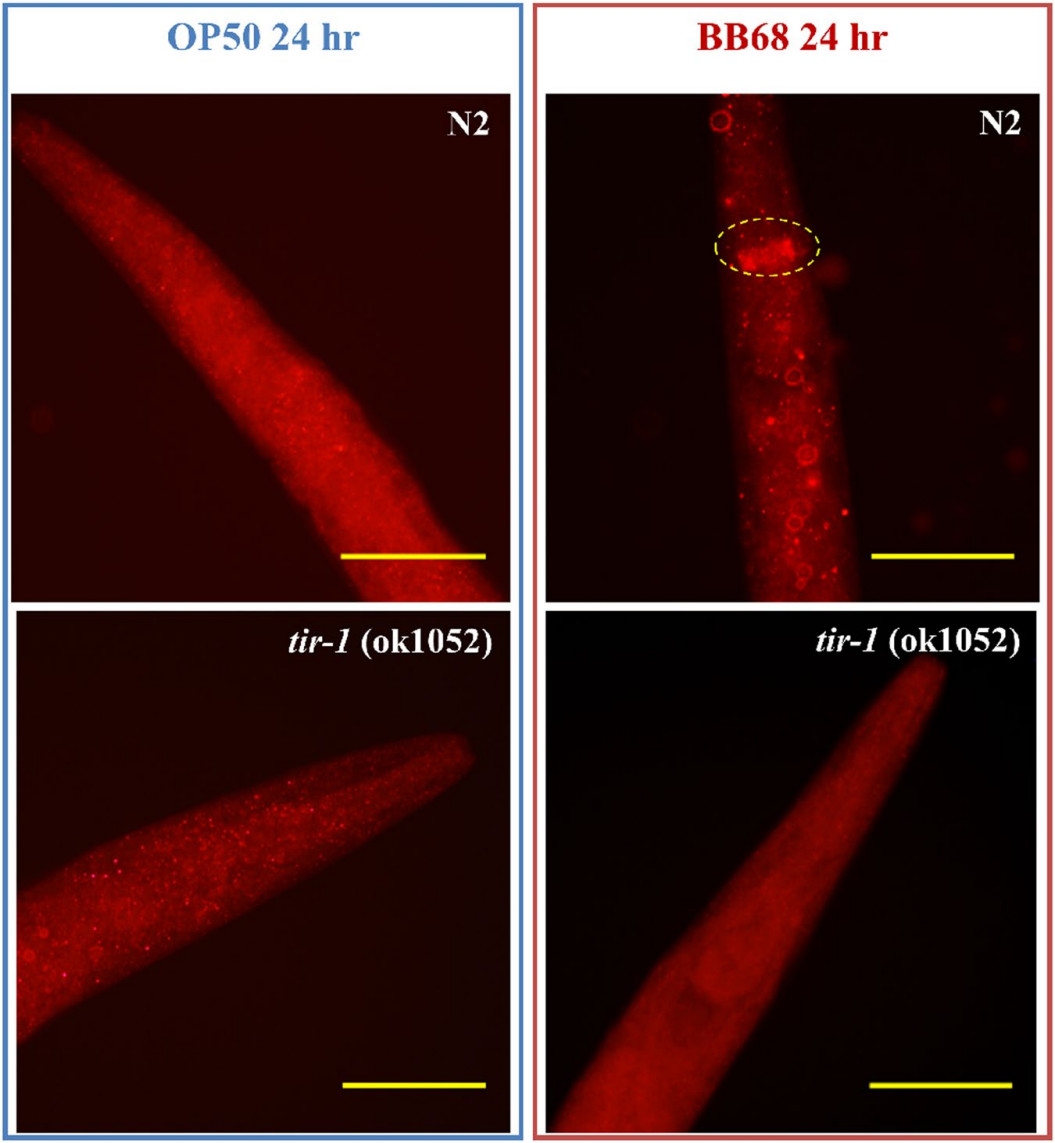
Feeding N2 worms BB68 for 24 h activated JNK-1 via phosphorylation. The phospho-JNK/SAPK (Thr183/Tyr185) antibody and Cy3-labeled goat anti-mouse IgG (H + L) were used as primary and secondary antibodies, respectively. The dashed circle indicates phosphorylated JNK-1 in the nerve ring, but no activated JNK-1 was detected in the presence of mutant *tir-1*.

In addition to functioning in ILS, DAF-16 is downstream of MAPK. To identify the regulator in the MAPK pathway involved in the BB68-induced, DAF-16-mediated regulation of the extension of lifespan, JNK and p38 were assessed. JNK-1 and PMK-1/p38, two members of the MAPK family in *C. elegans*, can transmit environmental stress signals to DAF-16 and regulate its nuclear localization^12, 13^. Our results show that BB68 extends the lifespan of *pmk-1* (km25) mutants, but not *jnk-1* (gk7) mutants (Table S1; Fig. 1E), and activated JNK-1 was observed in the nerve ring of BB68-treated nematodes (Fig. 2). In addition, the BB68-induced nuclear translocation of DAF-16 was decreased in worms with a mutated JNK-1 gene (Fig. 1B; Table S3). Thus, JNK appears to be involved in the BB68-mediated regulation of DAF-16. Additionally, we found that the TIR-domain protein TIR-1 participates in the BB68-mediated longevity signaling cascade. BB68 did not increase the lifespan of *tir-1* (ok1052) worms (Fig. 1E; Table S1), and mutations in TIR-1 suspended JNK-1 activation and BB68-induced DAF-16 nuclear accumulation (Table S3; Fig. 2). Therefore, we concluded that BB68 regulates DAF-16 through the TIR–JNK signal transduction pathway, an innate immunity signaling cascade conserved from nematodes to mammals.

**Figure 3:**
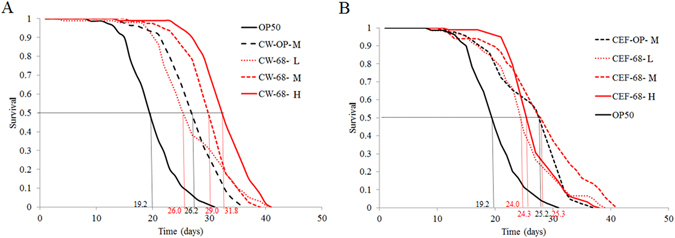
The cell wall is the main effective component of BB68 on nematode longevity. (**A**) Feeding worms the cell wall (CW) of BB68 (0.12 mg and 1.2 mg per plate) significantly increased the lifespan of nematodes relative to feeding them OP50 CW (0.12 mg per plate). (**B**) Feeding worms cell wall-free extracts (CFE) of BB68 did not affect the lifespan of nematodes relative to the lifespan of nematodes fed OP50 CFE.

To assess the potential active component of BB68 on nematode longevity, the cell wall component (CW) and cell wall-free extracts (CFE) of BB68 were separated and fed to nematodes. Compared to the CF or CFE of OP50, the CW of BB68 significantly increased the lifespan of *C. elegans* in a dose-response manner (*P* < 0.05); however, the CFE of BB68 did not exert a similar effect (Fig. 3). Thus, these results indicate that the cell wall components of BB68 contribute to its effects on longevity in nematodes.

**Figure 4:**
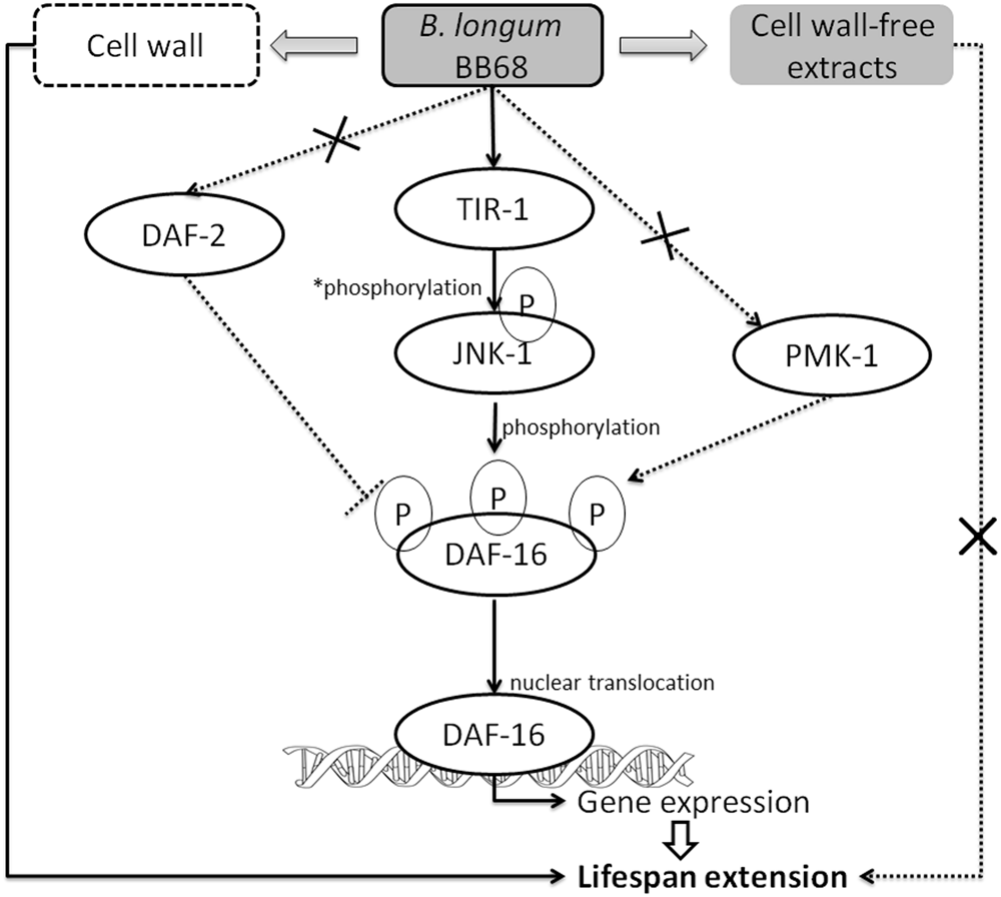
Mechanism by which BB68 affects the longevity of *C. elegans*. BB68 increased the longevity of nematodes by activating the TIR-1 – JNK-1 – DAF-16 signaling pathway, and the cell wall component of BB68 contributed to longevity. The solid arrow indicates activation observed in this study. A dashed line with an ‘×’ indicates that no effect was observed in this study. A dashed line indicates that the test was not performed in this study. A “P” indicates a phosphorylation site. *Indicates direct or indirect phosphorylation.

Our data confirm that *Bifidobacterium longum* strain BB68 prolongs the lifespan of *C. elegans* by regulating the activity of DAF-16, which is involved in a conserved immune signaling cascade (Fig. 4). Since these regulators are likely conserved across several species, the possibility of increasing the longevity of humans through the consumption of probiotics is elevated. Nevertheless, future studies are essential to determine the effect of *Bifidobacterium longum* BB68 on longevity in mammals.

## Methods

### Bacterial strains and culture conditions

*Bifidobacterium longum* strain BB68 was isolated from fecal samples of healthy centenarians in Bama County, Guangxi, China, and preserved in the laboratory. *E. coli* OP50 (OP50), a standard food for nematodes, was obtained from the *Caenorhabditis* Genetics Center (CGC, USA).

The BB68 strain was cultured at 37 °C in anaerobic conditions with GENbox anaer (bioMérieux, France) using MRS broth (LuQiao, China) for 18 h. OP50 was grown in LB broth (AoBoXing, China) at 37 °C for 18 h with agitation at 220 rpm. Bacteria were harvested by centrifugation at 4,000 × *g* for 15 min, washed twice with sterile M9 buffer, and centrifuged at 16,000 × *g* for 15 min at 4 °C to remove the supernatant. Then, the bacteria were adjusted to a final concentration of 0.4 mg/µL (wet weight) in M9 buffer; this slurry was used as concentrated bacteria and preserved at −80 °C. Before use, the concentrated bacteria were transferred into a 95 °C water bath for 30 min and used as heat-killed bacteria.

### Nematodes and growth conditions

*C. elegans* Bristol strain N2, *eat-2* (ad1116), *daf-16* (mu86), *daf-16* (mu86); muIs61, *daf-2* (e1368), *pmk-1* (km25), *jnk-1* (gk7), *tir-1* (ok1052) were obtained from CGC. The strains *jnk-1* (gk7); daf-16 (mu86); muIs61 and *tir-1* (ok1052); daf-16 (mu86); muIs61 were constructed according to Michael Koelle’s *C. elegans* protocols (http://medicine.yale.edu/lab/koelle/protocols/index.aspx). The primers are listed below.

*JNK1*F: 5′-GTAGAAGCGTGGAAGAGGA-3′; *JNK1*R: 5′-AGCTCTCCAAATATACACCC-3′. The wild-type amplicon is 1951 bp, and the *jnk-1* (gk7) amplicon is 765 bp.

*TIR1*F: 5′-AGATAAAGTCGGCAACCTGC; *TIR1*R: 5′-CAAATGGCGATCTGTACCCT. The wild-type amplicon is 3224 bp, and the *tir-1* (ok1052) amplicon is 1265 bp.

All *C. elegans* strains were maintained at 25 °C (unless otherwise stated) on nematode growth medium (NGM) agar supplemented with OP50 using standard techniques. For the lifespan assay, mNGM plates were produced by supplementing the NGM plates with filter-sterilized carbenicillin (0.5 mM) and 5-fluoro-2′-deoxyuridine (FUdR) (50 μM). Heat-killed bacteria (10 mg) were spread on 60 mm mNGM plates.

### Lifespan assay

L4 stage nematodes grown on NGM plates were transferred to mNGM plates with a platinum wire. For each lifespan assay, 100 worms for every bacterial species were assayed in ten plates (ten worms/plate), and plates were cultured at 25 °C. The number of live and dead worms was determined using a dissecting microscope (Chong Qing Optical, China) every 24 h. The lifespan assay was conducted at least three times. The nematode survival rate was calculated using the Kaplan–Meier method, and differences in survival rates were evaluated for significance using the log-rank test (*P* < 0.05).

### Food gradient feeding assay

Heat-killed BB68 and OP50 bacterial suspensions were spread on mNGM plates in serial concentrations ranging from 0.01–200 mg bacterial cells/plate. L4 stage N2 worms were placed on these plates. For each concentration, 100 worms were assayed over ten plates (ten worms/plate). Lifespan was measured as described previously. The test was conducted at least three times.

### Measurements of body size, pharynx pumping rate, and reproduction

L4 larvae were placed on NGM plates coated with bacterial lawns. Ten worms/bacterial species were assayed using ten plates (one worm/plate). Beginning on the first day that *C. elegans* were transferred to fresh NGM plates, the size of the live worms was examined every 24 h. Images of adult nematodes were captured using an XSP-8CZ digital microscope (Chong Qing Optical), and the projection area of the worms was analyzed as the body size using ImageJ software (National Institutes of Health, USA). To determine the pharynx pumping rate, L4 nematodes were grown on NGM plates in the presence of bacterial lawns for 30 min before counting. The pumping frequency was recorded as the number of contractions in the terminal bulb of the pharynx of an individual worm in a 60-s period. The experiment was performed five times. For the reproduction assay, the worms were transferred daily to fresh NGM plates until reproduction ceased. The offspring of each animal were counted at the L2 or L3 stage. The test was repeated three times.

### Determination of SOD-3 gene expression

Total RNA was extracted from L4 stage worms using TRNzol (Tiangen, China) accompanied by BeadBeater-16 (BioSpec, USA). Reverse transcription was completed with a Quantscript RT Kit (Tiangen). The expression of the *SOD-3* gene was detected using the primers *SOD3*F: 5′-GGTTGCGGGAGTTCTCGCCG-3′ and *SOD3*R: 5′-TCCCTTTCGAAACAGCCTCGTG-3′ on a Quantica Real-Time PCR system (Techne, UK). The *ACTIN-2* gene was used as an internal reference gene.

### DAF-16::GFP localization assay

L4 larvae of DAF-16::GFP worms were scored for nuclear, cytoplasmic, or intermediate fluorescent protein (GFP) localization as described by Oh *et al*.^12^. The number of worms with each level of nuclear translocation was determined. The percentage of worms showing nuclear and intermediate fluorescent translocation out of 100 worms was calculated as the DAF-16 nuclear location %. The assay was conducted five times.

### Nuclear and cytosolic distribution of DAF-16 (Western blotting)

The L4 larvae of DAF-16::GFP worms were collected. Subsequently, the nuclear and cytosolic fractions were separated, and the DAF-16 protein was detected by Western blotting as described by Singh and Aballay^14^. Lamin B1 and GAPDH were used as markers for the nuclear and cytosolic fractions, respectively. Antibodies against GAPDH, DAF-16, and lamin B1 were obtained from Cell Signaling Technology (CST; USA), Santa Cruz Biotechnology (USA), and Abcam (USA), respectively.

### Immunofluorescence staining assay

Immunofluorescence staining for phosphorylated-JNK-1 in L4 larvae was carried out according to Michael Koelle’s *C. elegans* protocols (http://medicine.yale.edu/lab/koelle/protocols/index.aspx). A phospho-JNK/SAPK (Thr183/Tyr185) antibody and Cy3-labeled goat anti-mouse IgG (H + L) (Beyotime Biotech, China) were utilized as primary and secondary antibodies, respectively. Fluorescence images were obtained with a ZEISS Axio Vert.A1 inverted microscope with excitation at 550 nm.

### Separation of CW and CFE of bacteria

OP50 and BB68 were cultured, and cells were harvested by centrifugation at 14,000 × *g* for 10 min at 4 °C. Then, the CW and CFE were separated by sonication as described by Komura *et al*.^15^. The CW and CFE were freeze dried and stored at −80 °C until further use. For the lifespan assay, CW and CFE were solubilized at 50 mg/mL in M9 buffer and added to heat-killed OP50 in a concentration gradient. Fifty microliters of the mixture was spread on 35 mm mNGM plates containing 3.4 mg of heat-killed OP50 and different concentrations of CW and CFE (0.012 mg, 0.12 mg, and 1.2 mg as low, medium, and high concentrations, respectively). Then, the lifespan assays were performed as described above. The medium concentration (0.12 mg) of CW or CFE from OP50 was used as a control in the lifespan assays.

## Electronic supplementary material

Supplementary Information
