# Supplementary material for: The Transcription Factor DAF-16 is Essential for Increased Longevity in C. elegans Exposed to Bifidobacterium longum BB68
Source: Sci Rep. 2017 Aug 7;7:7408. doi: 10.1038/s41598-017-07974-3 (PMC5547167; doi:10.1038/s41598-017-07974-3)
Supplement: Supplementary file 1 — Supplementary Information [file 41598_2017_7974_MOESM1_ESM.doc]

**Supplementary Information**

**The Transcription Factor DAF-16 is Essential for Increased Longevity in *C. elegans* Exposed to *Bifidobacterium longum* BB68**

Liang Zhao^1, 2^, Yang Zhao^2, 3^, Ruihai Liu^4^, Xiaonan Zheng^2, 3^, Min Zhang^5^, Huiyuan Guo^1, 2^, Hao Zhang^1, 3^, and Fazheng Ren^1, 2, 3, 6, *^

^1^ *Beijing Advanced Innovation Center for Food Nutrion and Human Health, College of Food Science and Nutritional Engineering, China Agricultural University, Beijing 100083, China.*

^2^ *Key Laboratory of Functional Dairy,* *College of Food Science and Nutritional Engineering,* *China Agricultural University, Beijing 100083, China.*

^3^ *Beijing Laboratory for Food Quality and Safety, China Agricultural University, Beijing 100083, China.*

^4^ *Department of Food Science, Cornell University, Ithaca, NY 14853-7201, USA*

^5^ *School of Food and Chemical Engineering, Beijing Technology and Business University, Beijing 100048, China*

*^6^ Beijing Higher Institution Engineering Research Center of Animal Product, College of Food Science and Nutritional Engineering, China Agricultural University, Beijing 100083, China.*

^*^ To whom correspondence should be addressed. E-mail: [renfazheng@263.net](mailto:renfazheng@263.net)

**Table S1. Changes of lifespans in *C. elegans*** treated with BB68.

| Genotype | Mean lifespan ± SD (n) (Day) | | *p* value ^*^ | Percent changes^**^ | Trials |
| --- | --- | --- | --- | --- | --- |
|  | OP50 feeding | BB68 feeding |  |  |  |
| N2 | 16.1 ± 1.9 (704) | 20.5 ± 2.4 (616) | < 0.001 | 28% | 6 |
| *eat-2* (ad1116) | 24.3 ± 0.9 (335) | 26.3 ± 1.5 (318) | 0.006 | 8% | 3 |
| N2^0.01^ | 15.9 ± 1.0 (196) | 17.2 ± 1.1 (199) | 0.382 | - | 2 |
| N2^0.1^ | 18.0 ± 0.9 (191) | 20.6 ± 1.1 (189) | 0.006 | 14% | 2 |
| N2^1^ | 19.7 ± 1.0 (200) | 22.1 ± 1.2 (195) | 0.004 | 12% | 2 |
| N2^100^ | 17.2 ± 1.0 (194) | 19.7 ± 1.3 (191) | 0.004 | 15% | 2 |
| N2^200^ | 18.1 ± 0.9 (196) | 19.0 ± 1.1 (190) | 0.158 | - | 2 |
| *daf-16* (mu86) | 11.5 ± 0.8 (315) | 10.6 ± 0.7 (292) | 0.004 | -8% | 3 |
| *daf-2* (e1368) | 25.8 ± 1.5 (422) | 30.0 ± 1.9 (398) | < 0.001 | 16% | 4 |
| *jnk-1* (gk7) | 15.0 ± 1.2 (294) | 15.4 ± 1.4 (290) | 0.275 | - | 3 |
| *tir-1* (ok1052) | 18.8 ± 1.3 (308) | 18.5 ± 1.2 (321) | 0.401 | - | 3 |
| *pmk-1* (km25) | 17.5 ± 1.1 (216) | 19.3 ± 1.4 (210) | 0.002 | 11% | 2 |

^*^ Kaplan-Meier Survival Analysis was employed. The differences between two bacterial strain treatments were compared by log rank tests (*P* < 0.05).

^**^ Only significant changes were calculated (*P* <0.05).

N2^0.01^, N2^0.1^, N2^1^, N2^100^ and N2^200^ indicates that treating *C. elegans* N2 with 0.01, 0.1, 1, 100 and 200 mg heat-killed bacteria per plate (diameter = 60 mm). The nematodes in other experimental groups were treated with 10 mg heat-killed bacteria per same plate.

**Table S2**. Changes of body size, brood size and pumping rate of nematodes treated with BB68.

|  | OP50 feeding | BB68 feeding | *p* value^*^ | Trials |
| --- | --- | --- | --- | --- |
| Body projected area  (n) mm^2^ | 0.063 ± 0.005 (45) | 0.058 ± 0.005 (45) | 0.193 | 3 |
| Brood size (n) | 112.6 ± 56.8 (45) | - 1. ± 19.1 (45) | 0.342 | 3 |
| Pumping rate  (n) pumping/min | 194.9 ± 21.5 (10) | 191.8 ± 54.6 (10) | 0.858 | 5 |

^*^ The difference between two bacterial strain treatments was compared by ANOVA with Duncan's test (*P* < 0.05).

**Figure S1. The bacterial gradient concentration feeding assay of BB68 and OP50.**

Feeding BB68 significantly increased the lifespan of nematodes under a wide range of bacterial concentration (0.1 mg/plate to 200 mg/plate). The highest lifespan of nematodes was obtained at bacterial concentration of 1.0 mg/plate, regardless feeding OP50 or BB68, which represented a CR manner. That indicated that 10 mg/plate of BB68 (experimental concentration in this study) could not induce CR. ** indicated significant differences between OP50 and BB68 (log rank tests, *P* < 0.01).

**Table S3. Changes of DAF-16 nuclear location treated with BB68.**

| Genotype | DAF-16 nuclear location (n) % | | *p* value^*^ | | Percent changes^**^ | | Trials |
| --- | --- | --- | --- | --- | --- | --- | --- |
|  | OP50 feeding | BB68 feeding |  |  |  |  |  |
| daf-16 (mu86); muIs61 | 42.4 ± 6.9 (198) | 66.5 ± 11.4 (157) | | < 0.001 | | 56% | 3 |
| *jnk-1* (gk7); *daf-16* (mu86); muIs61 | 51.3 ± 15.2 (124) | 46.8 ± 8.7 (156) | | 0.532 | | - | 3 |
| *tir-1* (ok1052); *daf-16* (mu86); muIs61 | 48.2 ± 9.5 (181) | 50.5 ± 13.5 (210) | | 0.361 | | - | 3 |

^*^ The difference between two bacterial strain treatments was compared by ANOVA with Duncan's test (*P* < 0.01).

^**^ Only significant changes were calculated (*P*<0.01).
